# Helicobacter pylori Infection Elicits Type I Interferon Response in Human Monocytes via Toll-Like Receptor 8 Signaling

**DOI:** 10.1155/2022/3861518

**Published:** 2022-10-22

**Authors:** Chalystha Yie Qin Lee, Yee Teng Chan, Yi Ying Cheok, Grace Min Yi Tan, Ting Fang Tang, Heng Choon Cheong, Jamuna Vadivelu, Suhailah Abdullah, Chung Yeng Looi, Won Fen Wong

**Affiliations:** ^1^Department of Medical Microbiology, Faculty of Medicine, University of Malaya, 50603 Kuala Lumpur, Malaysia; ^2^School of Health and Biomedical Sciences, RMIT University, Bundoora, VIC 3083, Australia; ^3^Institute of Systems, Molecular, and Integrative Biology, University of Liverpool, Liverpool L7 8TX, UK; ^4^Department of Medicine, Faculty of Medicine, University of Malaya, 50603 Kuala Lumpur, Malaysia; ^5^School of Bioscience, Taylor's University, 47500 Subang Jaya, Selangor, Malaysia

## Abstract

*Helicobacter pylori* colonization and persistence could precede gastric adenocarcinoma. Elucidating immune recognition strategies of *H. pylori* is therefore imperative to curb chronic persistence in the human host. Toll-like receptor 7 (TLR7) and TLR8 are widely known as viral single-stranded RNA (ssRNA) sensors yet less studied in the bacteria context. Here, we investigated the involvement of these receptors in the immunity to *H. pylori*. Human THP-1 monocytic cells were infected with *H. pylori*, and the expression levels of human Toll-like receptors (TLRs) were examined. The roles of TLR7 and TLR8 in response to *H. pylori* infection were further investigated using receptor antagonists. Among all TLR transcripts examined, TLR8 exhibited the most prominent upregulation, followed by TLR7 in the THP-1 cells infected with *H. pylori* J99 or SS1 strains. *H. pylori* infection-mediated IFN-*α* and IFN-*β* transactivation was significantly abrogated by the TLR7/8 (but not TLR7) antagonist. Additionally, TLR7/8 antagonist treatment reduced *H. pylori* infection-mediated phosphorylation of interferon regulatory factor 7 (IRF7). Our study suggests a novel role of TLR8 signaling in host immunity against *H. pylori* through sensing live bacteria to elicit the production of type I interferon.

## 1. Introduction

*Helicobacter pylori* is a *Gram*-negative bacterium that selectively colonizes the gastric epithelium of roughly 50% of the world's population [1]. The bacterium has a causal relationship with gastritis and gastric cancer [2]. The success of *H. pylori* lies in its life-long persistence in the host and exiguous host immune response towards its continuous presence. Because the bacterium has a long history of coexistence within human, it has coevolved with human and is capable of establishing a well-strategized equilibrium between bacterial effectors and host immune response [3–5]. One of the first lines of defense against *H. pylori* is the infiltration of monocytes and macrophages at the infected area to engulf extracellular bacteria and activate adaptive immune response [6, 7].

Human cells express pattern recognition receptors (PRRs) such as Toll-like receptors (TLRs) that detect distinct conserved constituents of microorganisms called pathogen-associated molecular patterns (PAMPs) to initiate inflammatory response [8]. Members of the TLR family in humans include TLR1 to TLR10, which recognize and bind to their respective ligands on the bacterial cell surface or internalized nucleic acids in intracellular vesicles [9]. Following *H. pylori* infection, several surface TLR signaling pathways are provoked for distinct inflammatory cytokine secretions [10]. For instance, infection of mouse bone marrow-derived macrophages (BMDM) with *H. pylori* stimulates the production of interleukin-6 (IL-6) and IL-1*β* via TLR2, IL-12, and IL-10 through the TLR4 pathways [11]. Attenuated IL-6 production was reported in peritoneal macrophages isolated from *H. pylori*-infected TLR2-, TLR4-, and MyD88-deficient mice [12]. Point mutation in the gene encoding TLR4 impaired NF-*κ*B activation and TNF-*α* secretion following *H. pylori* infection [13]; although, some studies suggest that TLR4 is less responsive to the bacterial lipopolysaccharide (LPS) [14, 15]. TLR5 also recognizes *H. pylori* flagellin and is crucial for IL-8 and TNF-*α* production in a cagPAI-dependent manner [16]. For intracellular TLRs, TLR9 has been reported to promote cyclooxygenase 2 (COX2) expression following *H. pylori* infection [17]. TLR7 plays a minimal role in cytokine production as insignificant IL-6 and IL-12 levels were reported in TLR2/4/7/9- versus TLR2/4/9-deficient dendritic cells [18].

To date, a study has reported that *H. pylori* phagocytosis contributes to increased TLR8 [19] but less is known about its function in the immunity to the bacterium. Thus, we hypothesized that TLRs are exploited by the bacterium as an immune evasion strategy to establish a foothold in the host. In this study, we examined the expression of all TLRs, and our results have pinpointed a role of TLR8 in eliciting type I interferon in the host immunity against *H. pylori*.

## 2. Materials and Methods

### 2.1. Bacteria and Cell Infection

*H. pylori* J99 strain was obtained from American Type Culture Collection (ATCC) [20], and Sydney strain 1 (SS1) was acquired from University of Western Australia [21]. The bacteria were subcultured every 3-5 days on chocolate agar plates supplemented with 10% hemoglobin in microaerophilic conditions at 37°C, 10% CO_2_ in a humidified incubator. Methods for *H. pylori* inactivation were adapted from the published protocols [22, 23]. Bacterial colonies were harvested in phosphate buffered saline (PBS) and reconstituted to a concentration of 1 × 10^8^ CFU/mL. For heat inactivation, bacterial suspension was inactivated at 56°C for 30 min. For formalin inactivation, formalin was added to the bacteria to a final concentration of 0.025 M for 18 hours at room temperature and washed in PBS. Inactivated bacteria were stored in aliquots at -20°C.

THP-1 human monocytic cells (ATCC) were maintained in RPMI 1640 media supplemented with 10% heat-inactivated fetal bovine serum (FBS), 1% HEPES, 1% sodium pyruvate, 1% minimum essential medium nonessential amino acids (MEM NEAA), and 0.05 mM 2-mercaptoethanol (Sigma-Aldrich, Steinheim, Germany). THP-1 cells were cultured in a humidified incubator with 37°C, 5% CO_2_. Before infection, *H. pylori* colonies were adjusted by spectrophotometric quantification and then added to THP-1 cells (1 × 10^6^ cells/mL) at a multiplicity of infection (MOI) of 10 for 16 hours unless stated otherwise.

### 2.2. RNA Isolation and Quantitative Real-Time PCR (qRT-PCR)

RNA was isolated from THP-1 cells using RNeasy Mini Kit (Qiagen) and reverse-transcribed using iScript Reverse Transcriptase Supermix system (Bio-Rad, Hercules, CA) according to respective manufacturer's protocols. cDNA was amplified using the SYBR-FAST Kit (Kapa Biosystem, Wilmington, MA) in a 10 *μ*L reaction consisting of 5 *μ*L Master Mix, 0.2 *μ*L each of forward and reverse primers (10 *μ*M), 0.2 *μ*L ROX low, 10 ng cDNA, and PCR-grade water. Primers for each gene target were designed (Table 1), and primers for IFN were as reported in [24]. The cycling conditions were 3 minutes of initial denaturation at 95°C, 40 cycles of 95°C (denaturation), and 60°C (annealing and extension) for 1 minute, followed by a final dissociation step in an Mx3000P System (Agilent Technologies, Santa Clara, CA). The data were analyzed using the comparative CT method (2^-∆∆CT^).

### 2.3. Western Blot

Western blot analysis was performed as described in [5]. Primary antibodies used included anti-IKK*α* (3G12), IKK*β* (D30C6), phospho-IKK*α*/*β* Ser176/180 (16A6), IRF3 (D6I4C), phospho-IRF3 Ser396 (D6O1M), IRF7 (D2A1J), phospho-IRF7 Ser477 (D7E1W), and *β*-actin (D6A8) (Cell Signaling Technology, Danvers, MA). Band intensities of the western blot data were quantified using ImageJ software and presented as mean ± SD from three reading obtained [25].

### 2.4. ODN-Mediated Gene Silencing

Short single-stranded synthetic oligodeoxynucleotides (ODNs) can inhibit the activation of TLRs [26, 27]. ODN20958 is a TLR7 antagonist, whereas ODN20959 is a TLR7/8 antagonist [28]. THP-1 cells were seeded at a density of 1 × 10^6^ cells/mL in a 6-well plate, and ODN20958 and ODN20959 were then added into the wells to a final concentration of 2.0 *μ*M and were incubated for 4 hours before *H. pylori* infection.

### 2.5. Phagocytosis Assay of FITC-Labeled H. pylori

*H. pylori* was labeled with fluorescein isothiocyanate (FITC) as described with modifications [29]. In brief, bacterial colonies were resuspended in 1 mL of 0.1 M sodium bicarbonate buffer pH 9.0 and adjusted to 1 × 10^8^ CFU/mL. A total amount of 0.1 mL of 10 mg/mL FITC in anhydrous dimethyl sulfoxide (DMSO) was added to bacteria suspension and incubated in the dark at room temperature for 30 minutes with end-over rotation. The labeled bacteria were then washed 4× with sterile PBS and stored in aliquots at −20°C. THP-1 cells were infected with FITC-labeled *H. pylori* at MOI 50 for 4 hours or MOI 10 for 16 hours. The cells were then harvested, stripped off extracellular bacteria (see Section 2.6.), and analyzed in a FACSCanto II flow cytometer (BD Biosciences, San Jose, CA).

### 2.6. Stripping of Extracellular Bacteria

After infection with FITC-labeled bacteria, THP-1 cells were harvested and washed three times in Hanks' Balanced Salt Solution. Residual extracellular bacteria were removed as previously described in [29]. Briefly, the cells were washed with 30% sucrose and centrifuged at 250 × g at 4°C for 8 minutes. Cell pellets were then resuspended in PBS 3% FBS.

### 2.7. Immunofluorescence Microscopy

THP-1 cells were incubated on 96-well plate precoated with 0.01% poly-L-lysine (Merck) at room temperature, in dark for 1 hour, and fixed with methanol. Cells were visualized, and images were captured using EVOS M5000 fluorescence microscope (Invitrogen, Carlsbad, CA).

### 2.8. Statistical Analysis

Statistical analyses were performed using unpaired two-tailed Student's *t*-test in GraphPad Prism 5 (2007) software. *P*-values that are less than 0.05 were considered statistically significant.

## 3. Results

### 3.1. H. pylori Infection Induces Expression of TLR Transcripts in THP-1 Cells

**Figure 1:**
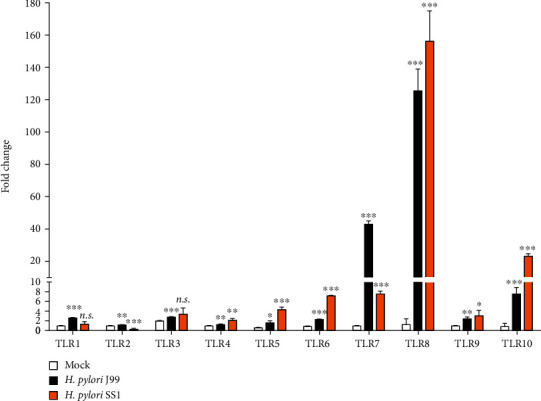
Upregulated expressions of *TLRs* in the THP-1 cells upon *H. pylori* infection. THP-1 cells were uninfected (mock) and infected with *H. pylori* J99 or SS1 strains at a MOI of 10 for 16 hours before RNA isolation and qRT-PCR analysis. *Y* axis shows the relative fold changes of the expression of the *TLR* transcripts relative to the *β*-*actin* endogenous housekeeping gene. Data is presented as mean ± SD from one experiment run in triplicate and is representative of two independent experiments. Statistical significance was analyzed using Student's *t*-test (n.s.: not significant; ^∗^*P* ≤ 0.05; ^∗∗^*P* ≤ 0.01; ^∗∗∗^*P* ≤ 0.001).

To examine the expression pattern of all *TLR* genes, two *H. pylori* strains, i.e., J99 (*CagA*^+^, *VacA s1m1*) and SS1 (*CagA*^+^, *VacA s2m2*) strains, were used to infect THP-1 cells at MOI of 10 for 16 hours. qRT-PCR results revealed that most mRNA transcripts for most of the *TLR* family members were upregulated upon exposure to *H. pylori* (Figure 1). Intriguingly, amongst all the *TLRs* studied, the expression of *TLR8* mRNA transcript was most prominently upregulated at 96.6-fold (*P* < 0.0001) and 120.5-fold (*P* = 0.0001) following infection by *H. pylori* J99 and SS1 strains, respectively, implying a potential role of TLR8 in immunity to *H. pylori*. TLR8 belongs to a group of endosomal TLRs that recognize nucleic acids. TLR8 and TLR7 recognize single-stranded RNA (ssRNA); other members of the endosomal TLRs include TLR3 that recognizes double-stranded RNA and TLR9 that recognizes hypomethylated CpG DNA [30]. Similar to *TLR8*, *TLR7* mRNA transcript was also increased remarkably at 44.2-fold (*P* < 0.0001) and 7.8-fold (*P* < 0.0001) upon J99 and SS1 infection. *TLR3* and *TLR9*, on the other hand, were only modestly upregulated. *TLR3* transcripts were increased marginally at 1.4- and 1.7-fold, while *TLR9* mRNA transcripts were increased at 2.4- and 3.1-fold, respectively, when infected with J99 and SS1 strains.

Another distinct group of cell surface plasma membrane-bound TLRs includes TLR2 homodimer which recognizes lipoprotein and peptidoglycans; respectively, TLR2/1 or TLR2/6 heterodimers recognize tri- or diacylated microbial lipopeptides [31]. TLR4 and TLR5, respectively, sense and respond to lipopolysaccharides and flagella, while the ligands for TLR10 remain unknown. Upon infection with *H. pylori* J99 strain, we noted that *TLR1*, *TLR2*, and *TLR6* mRNA transcripts were merely upregulated by approximately 2-fold. SS1 strain infection induced *TLR6* mRNA transcript up to 8.2-fold (*P* < 0.0001); however, the changes in expression levels of *TLR1* and *TLR2* were either insignificant or scarcely downregulated. The expression of *TLR4* mRNA transcripts was minimally affected at 1.3- and 2.2-fold when cells were infected with J99 and SS1 strains; in concordance with a previous study which reported that *H. pylori* LPS was unable to induce significant TLR4 response [15], *TLR5* transcripts were increased 2.7- and 7.4-fold upon infection with J99 and SS1, respectively. Interestingly, *TLR10* transcripts were elevated by 8.8-fold (*P* = 0.0009) and 27.4-fold (*P* < 0.0001) with J99 and SS1 infections. Previous report showed that *TLR10* was 3-fold higher in *H. pylori*-infected patients than *H. pylori*-negative participants [32]; however, its exact ligand and function require further investigations.

### 3.2. Induced TLR8 Protein Expression in H. pylori-Infected THP-1 Cells

**Figure 2:**
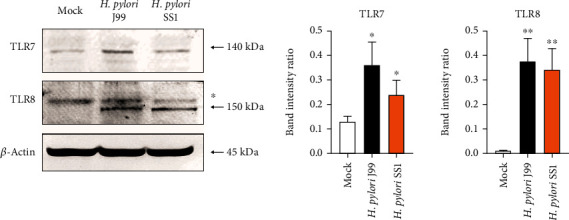
*H. pylori* induces TLR7 and TLR8 protein expression in THP-1 cells. Western blot analysis of protein lysates prepared from uninfected THP-1 cells (mock) or cells infected with *H. pylori* J99 and SS1 strains at a MOI of 10 for 16 hours. Membranes were probed with antibodies against TLR7, TLR8, and control *β*-actin. Asterisk (^∗^) indicates nonspecific band; arrow indicates bands of interest. (a) Western blot data. (b) Bar charts showing average ratio of TLR7 or TLR8 over *β*-actin band intensities. Data are representative of two independent experiments and shown as mean ± SD. Statistical significance was analyzed using Student's *t*-test (^∗^*P* ≤ 0.05; ^∗∗^*P* ≤ 0.01).

Both TLR7- and TLR8-mediated signaling pathways have mostly been implicated in antiviral activity through recognizing ssRNA. Interestingly, among all 10 *TLR* family members, we detected the highest fold change of *TLR8* transcript following exposure of THP-1 to *H. pylori* infection. These changes were further verified at protein level by immunoblot analysis (Figure 2). Both *H. pylori* J99 and SS1 strains were equally robust in promoting the expression of TLR8 protein. Consistent with the changes at the transcriptional level, *H. pylori* J99 infection was a stronger inducer of TLR7 protein compared to SS1 strain. A greater ability of J99 strain to induce TLR expression induction is attributable to the virulence gene *VacA s1m1* that is naturally associated with greater amounts of cytotoxin production and a higher risk of ulceration and gastric epithelial damage [20], while SS1 stain harbors *VacA s2m2* that is linked to weaker virulence in human cells [21].

### 3.3. TLR7 and TLR8 Induction in H. pylori-Infected THP-1 Cells Requires Live Bacteria

**Figure 3:**
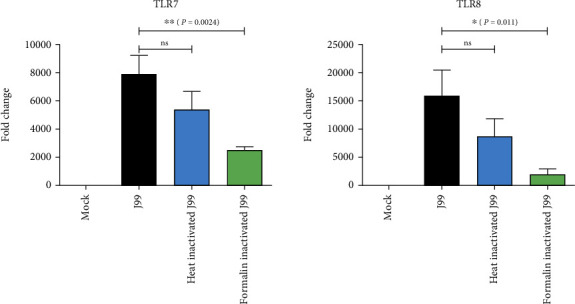
Expression levels of *TLR7* and *TLR8* transcripts in the THP-1 cells infected with live and death bacteria. THP-1 cells were uninfected (mock) or exposed to live, heat-, or formalin-inactivated *H. pylori* J99 strain at MOI 10 for 16 hours. The relative expression of *TLR7* and *TLR8* mRNA transcripts relative to the *β*-*actin* endogenous housekeeping gene. Statistical significance was analyzed using Student's *t*-test (^∗^*P* ≤ 0.05; ^∗∗^*P* ≤ 0.01; *ns*: not significant).

To further investigate if live bacteria are crucial for the *H. pylori*-mediated expression of TLR7 and TLR8, we infected the THP-1 cells with *H. pylori* J99 strain in three different forms, i.e., live, heat-, or formalin-inactivated. Our results showed that live bacteria were most efficient in the induction of both *TLR7* and *TLR8* transcripts (Figure 3). Induction of *TLR7* was 1.45-fold and 3.08-fold lower when the bacteria were killed by heat and formalin treatment, respectively, as compared with live bacteria. Similarly, *TLR8* was also attenuated by 1.8-fold in the heat-inactivated bacteria and 7.8-fold in the formalin-inactivated bacteria. This result suggests that, although the increment of fold-change were lower when infected with inactivated bacteria, both live and death bacteria could transactivate *TLR7* and *TLR8* genes. Hence, the presence of bacterial ssRNA is sufficient to trigger TLR7 and TLR8 activation, in the absence of live bacteria.

### 3.4. TLR7/8 Antagonist Abrogates H. pylori Infection-Mediated Transactivation of Type I Interferon

**Figure 4:**
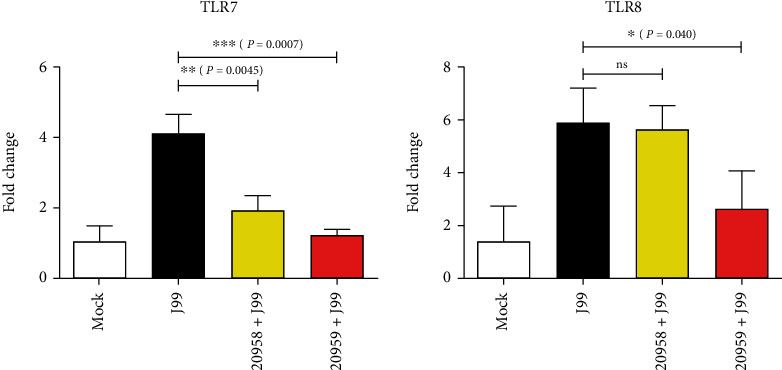
Effect of TLR7/8 antagonists on *TLR7* and *TLR8* expression THP-1 cells. THP-1 cells were uninfected (mock), infected with *H. pylori* J99 strain at MOI 10 for 16 hours (J99), or treated with TLR7 inhibitor (ODN20958 + J99) or TLR7/8 inhibitor (ODN20959 + J99) for 4 hours prior to infection. Expression levels of *TLR7* transcript were downregulated by both ODN20958 and ODN20959, whereas the expression of *TLR8* transcript was downregulated by ODN20959 alone, suggesting specificity of the antagonists. Data is presented as mean ± SD from one experiment run in triplicate and is representative of two independent experiments. Statistical significance was analyzed using Student's *t*-test (^∗^*P* ≤ 0.05; ^∗∗^*P* ≤ 0.01; ^∗∗∗^*P* ≤ 0.001; *ns*: not significant).

To further investigate the role of TLR7 and TLR8 in immunity to *H. pylori*, we disrupted the gene expression by introducing TLR7 antagonist (ODN20958) or TLR7 and TLR8 (TLR7/8) antagonist (ODN20959) [28]. Using these inhibitory ODNs, we can deduce if an effect was due to TLR7 or TLR8, either individually or synergistically. Prior treatment of cells with TLR7 and TLR7/8 antagonists before *H. pylori* infection resulted in a lower *TLR7* mRNA transcript expression to 2.14-fold (*P* = 0.0044) and 3.35-fold (*P* = 0.0007), respectively (Figure 4, left panel). Conversely, treatment with TLR7 antagonist ODN20958 demonstrated no significant effect on *TLR8*, whereas TLR7/8 antagonist ODN20959 reduced the *TLR8* mRNA transcript by 2.23-fold (*P* = 0.0404) (Figure 4, right panel).

**Figure 5:**
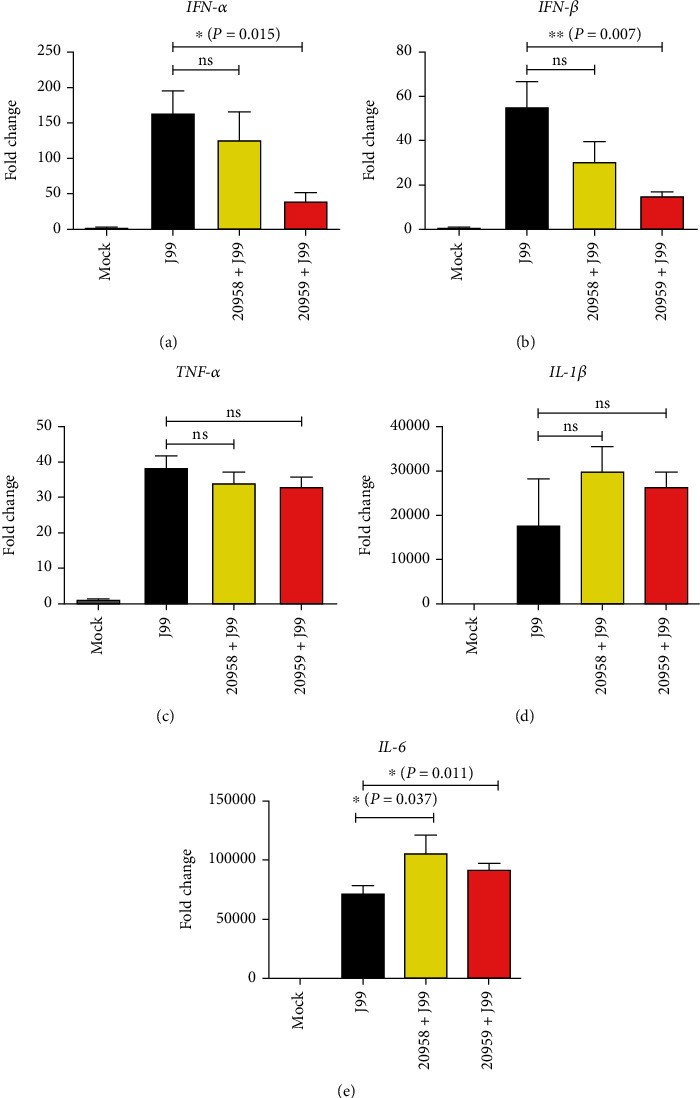
Effect of TLR7 and TLR8 antagonists on *H. pylori* infection-mediated inflammatory cytokine expression in THP-1 cells. THP-1 cells were uninfected (mock), infected with *H. pylori* J99 strain at MOI 10 for 16 hours (J99), or infected and treated with TLR7 inhibitor (ODN20958 + J99) or TLR7/8 inhibitor (ODN20959 + J99) for 4 hours prior to infection. Data is presented as mean ± SD from one experiment run in triplicate and is representative of two independent experiments. Statistical significance was analyzed using Student's *t*-test (^∗^*P* ≤ 0.05; ^∗∗^*P* ≤ 0.01; ^∗∗∗^*P* ≤ 0.001; *ns*: not significant).

Intracellular TLRs such as TLR7 and TLR8 are potent activators of type I interferon [33]. Since various cytokines in monocytes were induced following *H. pylori* infection, we further examined if cytokine production was disrupted when TLR7 and TLR8 signaling pathways were attenuated (Figure 5). Pretreatment of THP-1 cells with TLR7 antagonist ODN20958 prior to *H. pylori* infection marginally reduced the level of *IFN*-*α* mRNA transcript to 1.28-fold and *IFN*-*β* mRNA transcript to 1.84-fold, but both reductions were not statistically insignificant. Notably, pretreatment with TLR7/8 antagonist ODN20959 demonstrated a strong suppressive effect on the expression on both *IFN*-*α* and *IFN*-*β* mRNA transcripts to 4.2-fold (*P* = 0.0158) and 3.6-fold (*P* = 0.0070), respectively. No noticeable changes were observed when the transcription levels of *TNF*-*α* and *IL*-1*β* inflammatory cytokines were examined. In contrast, both TLR7 and TLR7/8 antagonists augmented the expression of *IL*-6 transcript to 1.5-fold (*P* = 0.0374) and 1.3-fold (*P* = 0.011). Although the changes of type I IFN cytokines were observed in the mRNA transcript level, attempt to detect IFN-*α* and IFN-*β* cytokine production in culture supernatant using ELISA assay had failed due to minimal secretory level of these cytokines below detection limit.

### 3.5. Phagocytic Function of THP-1 Cells Is Not Altered by TLR7/8 Antagonist

**Figure 6:**
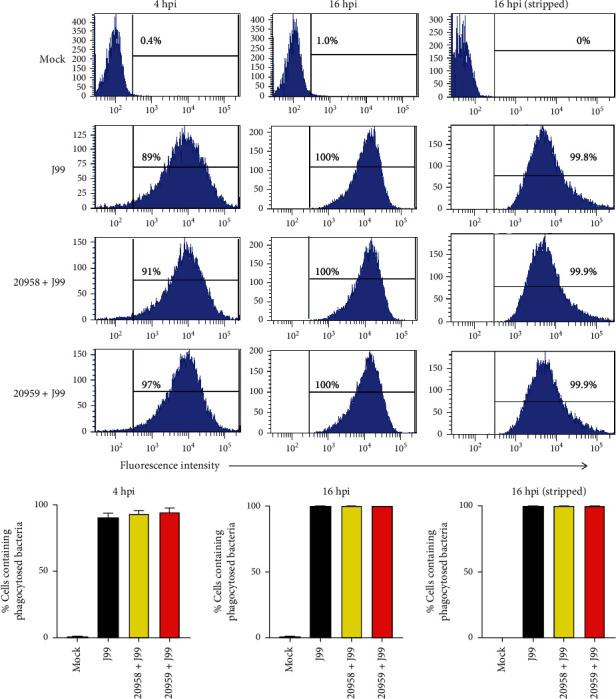
Phagocytic function of THP-1 macrophage is unaltered upon inhibition of both TLR7 and TLR8 mediated signaling. THP-1 cells were uninfected (mock), infected with *H. pylori* J99 strain, or infected and treated with TLR7 inhibitor (ODN20958 + J99) or TLR7/8 inhibitor (ODN20959 + J99), at MOI 50 for 4 hours or MOI 10 for 16 hours. Some cells were stripped off extracellular bacteria before analysis. Fluorescent intensity was examined by flow cytometrical analysis. (a) Histogram showing fluorescent intensity. (b) Bar charts showing the average percentages of cells containing FITC-labeled bacteria. Data is representative of three independent experiments.

To investigate whether TLR7 and TLR8 interference ablates the phagocytic ability of monocytes, we labeled *H. pylori* with FITC before adding to the THP-1 cells at an MOI of 10 for 4 or 16 hours and quantified the number of cells with internalized bacteria using flow cytometer. At 4 h.p.i., 89% of THP-1 cells exhibited fluorescence, indicating active phagocytosis of bacteria. At 16 h.p.i., all cells (100%) were fluorescent and exhibited higher degree of mean fluorescence intensity. THP-1 cells treated with TLR7 and TLR7/8 antagonists demonstrated no defect in bacteria uptake when examined using FITC-labeled bacteria followed by flow cytometry detection (Figure 6), indicating that the monocyte phagocytic activity was not affected by TLR8 signaling interference.

**Figure 7:**
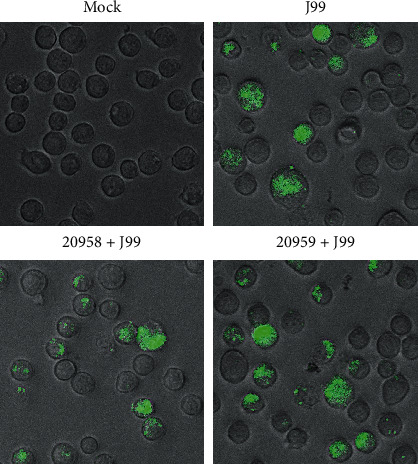
Unperturbed bacteria internalization in THP-1 macrophage with or without TLR7 and TLR8 inhibition. THP-1 cells were uninfected (mock), infected with *H. pylori* J99 strain conjugated with FITC at MOI 10 for 16 hours, or infected after treatment with TLR7 inhibitor (ODN20958 + J99) or TLR7/8 inhibitor (ODN20959 + J99) for 4 hours. Cells were stripped off extracellular bacteria and viewed under fluorescent microscope. Data are representative of at least 10 images captured.

To exclude the possibility that the *H. pylori* measurement was affected by the bacteria that were attached on cell surface, we removed the extracellular bacteria through stripping method before measurement [29]. We observed no different in the percentages of cells with FITC signal, before or after stripping, as shown in the flow cytometry data (Figure 6). Internalized bacteria can be clearly visualized under fluorescent microscopy (Figure 7).

### 3.6. TLR7/8 Antagonist Abrogates H. pylori Infection-Mediated IRF7 Phosphorylation

**Figure 8:**
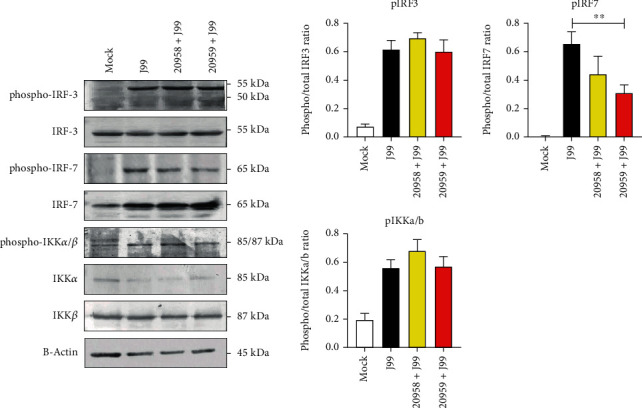
Immunoblot analysis of TLR downstream signaling pathway molecules. THP-1 cells were infected with *H. pylori* J99 strain at MOI 10 for 16 hours. Cells were lysed and processed for immunoblot analysis with antibodies to phospho- or total IRF-3, IRF-7, IKK*α*/*β*, and *β*-actin as loading control. (a) Western blot data. (b) Bar charts showing average ratio of phosphorylated over total protein after normalizing to the *β*-actin level. Data is representative of two independent experiments. Statistical significance was analyzed using Student's *t*-test (^∗∗^*P* ≤ 0.01).

IRF3 and IRF7 are two structurally homologous proteins of the IRF family that are critical in antiviral immunity through transmitting endosomal TLR signal to activate type I IFN [34]. IRF3 is ubiquitously expressed, whereas the IRF7 expression is mainly limited to plasmacytoid dendritic cells [35]. In addition to IRFs, canonical I*κ*B kinase (IKK) complex is also activated through MyD88 in all TLR activation except for TLR3. Activation of IKK promotes nuclear translocation of transcription factors nuclear factor-*κ*B (NF-*κ*B) that in turn induces the expression of multiple cytokines including TNF, IL-6, and IL-1*β*. To examine the signaling pathway in *H. pylori* infection, immunoblot analysis was performed (Figure 8). Total amount of IFR3 was not affected, whereas IRF7 was increased following *H. pylori* J99 infection. Notably, phosphorylation of IRF7, but not IRF3, was attenuated by treatment with TLR7/8 antagonist. On the other hand, phosphorylation of IKK*α*/*β* was unaffected by treatment with TLR7/8 antagonist.

## 4. Discussions

**Figure 9:**
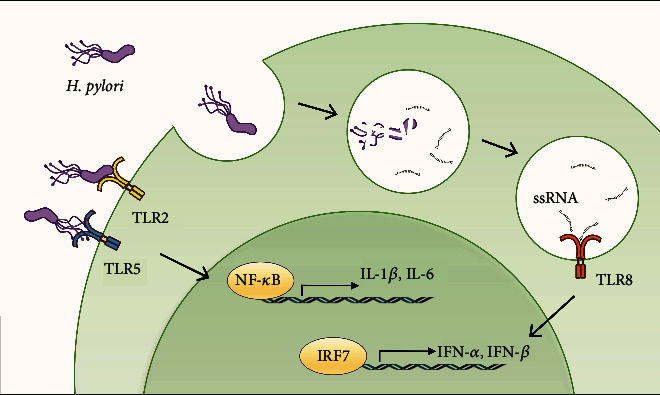
Illustration depicting the detection of *H. pylori* ssRNA by the endosomal TLR8 and downstream signaling pathway that results in transactivation of *IFN-α* and *IFN*-*β*. In contrast, extracellular bacteria molecules are detected mainly by TLR2 and TLR5 that trigger activation of NF-*κ*B that transactivates proinflammatory cytokines such as *IL-1β* and *IL-6*.

*H. pylori* infection triggers activation of the innate immune system in the host through recognition by multiple PRRs including TLRs [36]. Although both TLR7 and TLR8 are well established as viral RNA sensors, the role of TLR8 in bacterial RNA recognition has only begun to be uncovered in the past decade [37]. TLR7 is mainly expressed by plasmacytoid dendritic cells and is crucial for host production of type I interferon during viral infection [38]. On the other hand, TLR8 is primarily expressed in monocytes, macrophages, and dendritic cells. TLR8 receptor was reported to be induced in THP-1 macrophage by *Borrelia burgdorferi* [39], *H. pylori* [19], and *Mycobacterium tuberculosis* [40]. In the past, TLR8 was regarded as either nonfunctional or associated with recognition of viral ssRNA in the endosome; however, recent evidence have demonstrated that it senses bacterial RNA to induce expression of type I interferons [37]. Using inhibitory ODN specifically targeting TLR7 and TLR8, we demonstrated that the TLR7/TLR8 antagonist ODN20959 significantly diminished the production of *IFN*-*α* and *IFN*-*β* mRNA transcripts in *H. pylori*-stimulated monocytes. TLR7 antagonist alone exerted minimal but nonsignificant reduction of *H. pylori* infection-induced *IFN*-*α* and *IFN*-*β*. This result underscores the role of TLR8 but not TLR7 in the transactivation of type I interferon (Figure 9).

In our attempt to quantify IFN-*α* and IFN-*β* secretion levels, we noticed both were at low concentrations below detection limit of ELISA assay (data not shown). The expression levels of *IL*-1*β* and *IL*-6 were significantly increased following *H. pylori* infection at a greater extent than that of the type I interferon cytokines (Figure 5), indicating a predominantly inflammatory response during *H. pylori* infection. The relative expression of type I interferon *IFN*-*α* and *IFN*-*β* mRNA transcript was increased at approximately 60- to 150-fold following infection, while *IL*-1*β* and *IL*-6 recorded increase fold changes of more than 20,000- and 70,000-fold, respectively. Hence, it is not surprising that the level of IFN-*α* and IFN-*β* was not detectable in our study. We were unable to further optimize the cytokine production as an increased of bacteria MOI resulted in cell death of THP-1 cells. Perhaps, an *in vivo* infection model or clinical samples from *H. pylori*-infected individuals can be utilized in the future to quantify the secretory level of type I interferon following *H. pylori* infection.

Although the mRNA transcript levels of *IFN*-*α* and *IFN*-*β* were weakened in the presence of TLR8 antagonists, we noted that the exposure to TLR7 or TLR8 antagonists did not alter the transcriptions of the proinflammatory cytokines *IL*-1*β* and *TNF*-*α* but increased *IL*-6. This may be explained by the immunomodulatory effect of type I interferons, which favors the development of anti-inflammatory response. Correspondingly, TLR9 activation, which triggers type I interferon production, is suppressive to inflammatory response as higher degree of inflammation augmented by either IL-17 or T helper 1 responses was demonstrated in *H. pylori*-infected TLR9 knockout mice [41, 42]. TLR10, although shows correlation with *H. pylori* infection in ours and previous finding [32], is not further investigated here due to limited information on ligand and inhibitor.

It is known that bacterial RNA liberated from phagocytosed bacteria can trigger the activation of endosomal TLRs and elicit transcription of inflammatory cytokines and type I interferons through NF-*κ*B or IRF signaling pathways [43]. Both TLR7 and TLR8 recognize ssRNA of pathogen through different binding motifs. TLR7 has been reported to recognize guanosine and uridine-containing ssRNA [44], whereas TLR8 recognizes UR/URR RNA-ligand consensus motif [45]. Some bacteria such as *Escherichia coli* and *Thermus thermophilus* exploit RNA modifications to circumvent host immune activation [46, 47]. Interestingly, 2′-O-ribose methylation in the ribosomal RNA converts RNA from TLR7/TLR8 ligand into an exclusive TLR8 ligand [48]. This highlights the functional incongruities between TLR7 and TLR8, which explains the selective activation of TLR8 by *H. pylori* in our study. In fact, human TLR8 inhibits TLR7 and TLR9 signaling [44], whereas TLR8 deficiency demonstrates TLR7 overexpression and NF-*κ*B activation in mice, and is associated with the occurrence of autoimmune disorders in human [49, 50].

The downstream signaling pathways of TLRs lead to robust initiation of innate immune response. In the case of *H. pylori* infection, persistent stimulation led to increased expression and activation of TLRs, which has been reported to result in the progression of gastric ulcer and gastric cancer [51]. For instance, *H. pylori*–induced gastritis patients showed higher expressions of TLR2, TLR4, and TNF–*α* in their gastric biopsies of while metaplasia and dysplasia patient samples exhibited higher TLR2 expression [52]. TLR9 has been associated with tumorigenesis at late stage of gastric cancer [31] but it appears to play a minimal role in cytokines secretion as shown in an infected knockout mouse model [12]. This also highlights the potential involvement of PRRs in cancer development as more work is emerging to bridge the gap between cancer and inflammation.

The overall upregulated trend in TLR expression is supported by the finding of an increase in TLR2, TLR4, TLR5, and TLR9 expressions in the gastric epithelium of *H. pylori*-infected children. This increase in expression was also associated with an increase in the expression of pro- and anti-inflammatory cytokines, such as TNF-*α* and IL-10 [53], whose competition could establish a balance that allows the bacteria to persist in the host for decades and promote neoplastic transformation. TLR2 signaling following exposure to *H. pylori* is important for the production of IL-1*β*, IL-6, IL-8, and TNF-*α* to generate an inflammatory response [11, 15, 16]. Our results are in agreement with previous reports which showed upregulation of TLR5 following *H. pylori* infection [15, 16].

IRF family members including IRF3 and IRF7 play crucial role to trigger transactivation of type I IFN downstream of TLR signal [54]. These molecules share high homology in their structure and execute their functions through homodimer or heterodimer formation [55]. Similar to our finding, a previous study has shown that TLR8 is self-amplifying and induces secretion of IFN-*β* through IRF7 signaling pathway [39]. As a pathogen that coexisted with humans since ancient history, *H. pylori* has evolved many different strategies to evade immune attack and survive in the hostile environment of the host stomach [56, 57]. Hence, from our findings, we anticipate that TLR8-mediated induction of IRF7 signaling pathway counteracts the inflammatory reaction directed by TLR2- and TLR5-mediated signaling. Further studies using human samples would be interesting to investigate a correlation of type I IFN with the disease severity of chronic *H. pylori* infection.

## 5. Conclusion

In conclusion, TLR8 is identified as the most significantly upregulated molecule among all TLR members, which plays an indispensable role in secretion of type I interferon from macrophages in immunity against *H. pylori* infection.

## Figures and Tables

**Table 1:** Primer sequences used in real-time qRT-PCR analysis.

Target	Forward primer sequence (5′ to 3′)	Reverse primer sequence (5′ to 3′)	NCBI accession no.
*β*-Actin	GGA CGA CAT GGA GAA AAT CTG GCA	ACT CGT CAT ACT CCT GCT TGC TG	NM_001101.4
*TLR1*	GCC AAA TGG AAC AGA CAA GCA	ACA GAT TCC TTT TGT AGG GGT GC	NM_003263
*TLR2*	GTT GTG GGT TGA AGC ACT GG	GGC TTG AAC CAG GAA GAC GA	NM_003264
*TLR3*	TCA GAA GAT TAC CAG CCG CC	TGA AAA CAC CCT GGA GAA AAC TCT	NM_003265
*TLR4*	CAG AAT GCT AAG GTT GCC GC	TTA GGA ACC ACC TCC ACG C	NM_138554
*TLR5*	TTT CAG GAG CCC GAG CGA	ATA GCA TCC CTG GTT TGG TGA C	NM_003268
*TLR6*	ATG ACC AAA GAC AAA GAA CCT ATT G	AAT TCC TTA CAG ATG GGC AGG	NM_006068
*TLR7*	ACT CCA TGC CAT CAA GAA AGT TGA	TCT GTG CAG TCC ACG ATC AC	NM_016562
*TLR8*	GAA ACA TGG TTC TCT TGA CAC TTC A	CTG GTG CTG TAC ATT GGG GT	NM_016610
*TLR9*	GAA GGG GTG AAG GAG CTG TC	CGG CAG AAA CCC ATG CTG	NM_017442
*TLR10*	AAG CCG TGG GAA TTC AGC AG	TGC CAT GTC TCC GTT GTT GA	NM_030956
*IL-1β*	ATG CAC CTG TAC GAT CAC TG	ACA AAG GAC ATG GAG AAC ACC	NM_000576.3
*IL-6*	CCA CTC ACC TCT TCA GAA CG	CAT CTT TGG AAG GTT CAG GTT G	NM_000600.5
*TNF-α*	ACT TTG GAG TGA TCG GCC	GCT TGA GGG TTT GCT ACA AC	NM_000594.4
*IFN-α*	GAC TCC ATC TTG GCT GTG A	TGA TTT CTG CTC TGA CAA CCT	NM_024013.3
*IFN-β*	TGC TCT CCT GTT GTG CTT CTC	CAT AGA TGG TCA ATG CGG CG	NM_002176.4

## Data Availability

The data presented in this study are available on request from the corresponding author.
